# The deubiquitylase USP15 regulates topoisomerase II alpha to maintain genome integrity

**DOI:** 10.1038/s41388-017-0092-0

**Published:** 2018-02-12

**Authors:** Andrew B. Fielding, Matthew Concannon, Sarah Darling, Emma V. Rusilowicz-Jones, Joseph J. Sacco, Ian A. Prior, Michael J. Clague, Sylvie Urbé, Judy M. Coulson

**Affiliations:** Cellular and Molecular Physiology, Institute of Translational Medicine, University of Liverpool, Liverpool, UK

## Abstract

Ubiquitin-specific protease 15 (USP15) is a widely expressed deubiquitylase that has been implicated in diverse cellular processes in cancer. Here we identify topoisomerase II (TOP2A) as a novel protein that is regulated by USP15. TOP2A accumulates during G2 and functions to decatenate intertwined sister chromatids at prophase, ensuring the replicated genome can be accurately divided into daughter cells at anaphase. We show that USP15 is required for TOP2A accumulation, and that USP15 depletion leads to the formation of anaphase chromosome bridges. These bridges fail to decatenate, and at mitotic exit form micronuclei that are indicative of genome instability. We also describe the cell cycle-dependent behaviour for two major isoforms of USP15, which differ by a short serine-rich insertion that is retained in isoform-1 but not in isoform-2. Although USP15 is predominantly cytoplasmic in interphase, we show that both isoforms move into the nucleus at prophase, but that isoform-1 is phosphorylated on its unique S229 residue at mitotic entry. The micronuclei phenotype we observe on USP15 depletion can be rescued by either USP15 isoform and requires USP15 catalytic activity. Importantly, however, an S229D phospho-mimetic mutant of USP15 isoform-1 cannot rescue either the micronuclei phenotype, or accumulation of TOP2A. Thus, S229 phosphorylation selectively abrogates this role of USP15 in maintaining genome integrity in an isoform-specific manner. Finally, we show that USP15 isoform-1 is preferentially upregulated in a panel of non-small cell lung cancer cell lines, and propose that isoform imbalance may contribute to genome instability in cancer. Our data provide the first example of isoform-specific deubiquitylase phospho-regulation and reveal a novel role for USP15 in guarding genome integrity.

## Introduction

Ubiquitylation is a reversible post-translational modification that can target proteins for degradation or regulate their activity or cellular localisation [1]. Monoubiquitin or polyubiquitin chains are appended to substrates by E1/E2/E3 ligases, and may subsequently be removed by a family of almost 100 deubiquitylases (DUBs) to reverse signals or stabilise proteins [2, 3]. As specific substrates are gradually assigned to each DUB [4–6], it is becoming apparent that many play roles in cell cycle progression and maintenance of genome integrity [7–10]. DUBs can be regulated by conformational changes, adaptor proteins, or post-translational modifications, which control their activity or recruitment to specific complexes [11, 12]. In particular, phosphorylation may regulate the localisation, stability, or substrate interactions of DUBs [12, 13]. For example, during the cell cycle, periodic phosphorylation activates USP16 and USP37 [14, 15] but inactivates USP8 through recruitment of 14-3-3 proteins [16]. The regulated expression of DUBs may also control their cellular availability, and alternative splicing can generate DUB isoforms that are targeted to distinct subcellular compartments, as described for USP33 [17], or exhibit different substrate specificity, as recently suggested for ubiquitin-specific protease 15 (USP15) [18].

USP15 is a widely expressed DUB [19] that regulates diverse cellular processes. Importantly, USP15 copy number gains have been reported in glioblastoma, breast, and ovarian cancers [20] and copy number losses identified in pancreatic cancer [21]. The proposed targets for USP15 include numerous cancer-associated proteins and signalling pathways, such as the human papilloma virus E6 oncoprotein [22], adenomatosis polyposis coli (APC) tumour suppressor [23], nuclear factor of kappa light polypeptide gene enhancer in B-cells inhibitor alpha (IκBα) [24], pro-apoptotic caspase-3 [25], the transforming growth factor beta receptor [20], and its receptor-regulated SMAD (R-SMAD) effectors [26], p53 [27], human homolog of mouse double minute 2 (MDM2) [28] and the ubiquitin E3 ligase BRCA1-associated protein (BRAP) associated with the Ras-MAPK signalling cascade [29]. USP15 substrates include both polyubiquitylated and monoubiquitylated proteins. In the case of BRAP, USP15 reverses polyubiquitination promoting its stability [29], whereas USP15 removes monoubiquitin from R-SMADs enhancing their transcriptional activity [26]. A systematic interaction study revealed prominent association of USP15 with RNA-binding proteins and splicing factors [30], and USP15 depletion affects CRAF transcript levels [29].

These diverse targets and modes of action for USP15 suggest that its activity must be tightly regulated and directed within cells. Although USP15 predominantly localises to the cytoplasm [31], it performs specific functions in the nucleus [32], and at mitochondria [33] or polysomes [34]. Mechanisms to control USP15 activity within cells are suggested by evidence that USP15 is alternatively spliced [18, 35] and can be ubiquitylated or phosphorylated [29, 34, 36–39]. Despite these insights, it remains unclear how the multifarious cellular functions of USP15 are directed and regulated.

We recently discovered that USP15 controls stability of the RE1-silencing transcription factor (REST), a context-dependent tumour suppressor or oncogene, which is acutely degraded at mitosis [40] before being rapidly replenished in early G1 in a USP15-dependent manner [34]. We also observed that USP15 is regulated during the cell cycle. USP15 increases in abundance and becomes phosphorylated at the start of mitosis, coinciding with mitotic degradation of REST. Subsequently, USP15 is dephosphorylated in early G1 as REST re-accumulates [34]. Thus, USP15 can exhibit temporal activity during the cell cycle to promote accumulation of a specific protein. As pervasive mitotic phosphorylation often inactivates protein functions [37], USP15 phosphorylation may abrogate its role in stabilising REST at mitosis, allowing REST to degrade.

Here we set out to investigate the importance of alternative splicing and phosphorylation in regulating the functions of USP15 during the cell cycle. We identify topoisomerase II (TOP2A) as a novel protein that is regulated by USP15. Depletion of USP15 leads to a failure of TOP2A to accumulate as cells approach mitosis, and the subsequent formation of anaphase chromosome bridges, which fail to resolve leading to micronuclei. We find that both USP15 isoforms localise to the nucleus at prophase, and that USP15 isoform-1 is selectively phosphorylated on S229 at mitotic entry. Intriguingly, the role of USP15 in maintaining genomic stability through TOP2A is abrogated by S229 isoform-1 phosphorylation. As USP15 isoform-1 can be selectively upregulated in lung cancer cell lines, we propose this isoform imbalance may contribute to genome instability in cancer.

## Results

### USP15 promotes accumulation of TOP2A during G2

**Fig. 1:**
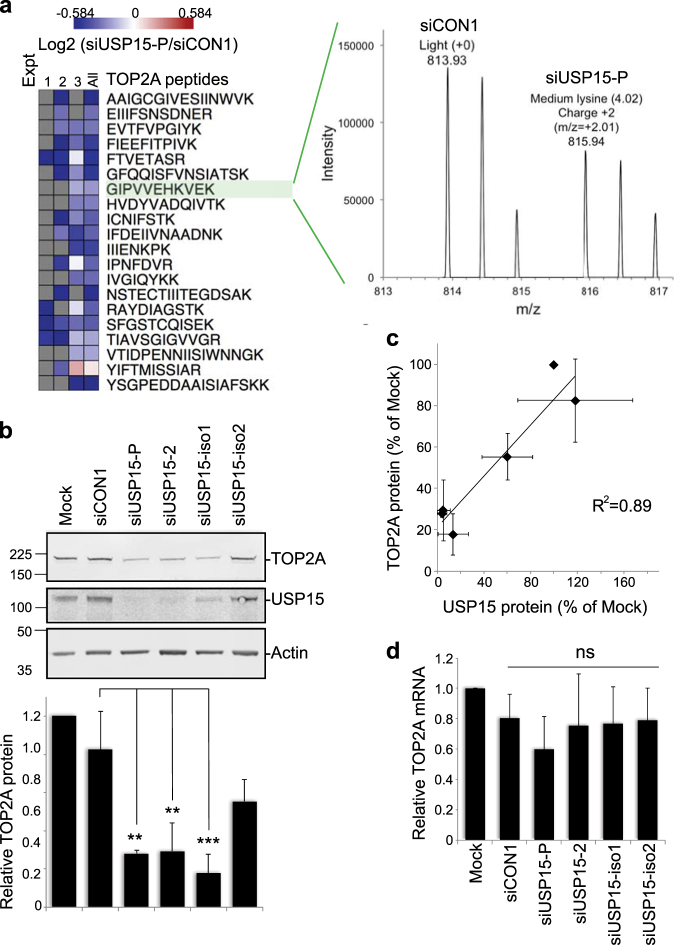
USP15 regulates the cellular level of TOP2A. **a** TOP2A was identified from a data set of proteins that differed in abundance on USP15 depletion. SILAC-labelled A549 cells were transfected with pooled siRNA targeting USP15 or a non-targeting control (siCON1) for mass spectrometry analysis. Multiple TOP2A peptides were downregulated in USP15 depleted cells across three independent experiments (left); spectra for an example peptide shown (right). **b**, **c** TOP2A protein expression correlates with total USP15 levels. A549 cells were transfected with siRNAs as indicated and whole-cell lysates analysed 72 h later. **b** Representative immunoblot, with quantification of TOP2A protein expression relative to actin below (mean of three independent experiments, error bars SD, one-way ANOVA with Tukey’s multiple comparison test **P ≤ 0.01, ***P ≤ 0.005). **c** Correlation of TOP2A protein and total USP15 protein in these experiments. **d** TOP2A mRNA level was not affected by USP15 depletion. qRT-PCR analysis of TOP2A relative to actin (mean of four independent experiments, error bars SD, ns = not significant by one-way ANOVA with Tukey’s multiple comparison test)

Two ~110 kDa isoforms of USP15 have until recently been interchangeably studied in the literature. These isoforms arise through alternative splicing that skips (isoform-2) or includes (isoform-1) exon 7, coding for a serine-rich cassette of 28 amino acids (Supplementary Figure S1a). We found that A549 lung adenocarcinoma cells express both USP15 isoforms, with higher levels of isoform-1, which can be distinguished by isoform-specific small interfering RNAs (siRNAs) and reverse transcription-PCR (RT-PCR) primers (Supplementary Figure S1). We wished to identify new candidates that may respond to dynamic USP15 regulation during the cell cycle. To this end, we interrogated a quantitative mass spectrometry data set of protein abundance in response to USP15 depletion in A549 cells (Concannon et al., unpublished data) for likely candidates. We identified TOP2A as a novel protein that was consistently downregulated by a pool of USP15 siRNA (Fig. 1a). We confirmed the relationship between USP15 and TOP2A expression levels by immunoblotting; TOP2A decreased significantly on depletion of total USP15, or of the most abundant isoform-1 (Fig. 1b). Overall, there was a strong correlation between residual USP15 and TOP2A proteins across all samples (Fig. 1c), suggesting both USP15 isoforms can function to maintain TOP2A levels. Of note, USP15 depletion did not significantly alter TOP2A mRNA expression (Fig. 1d).

**Fig. 2:**
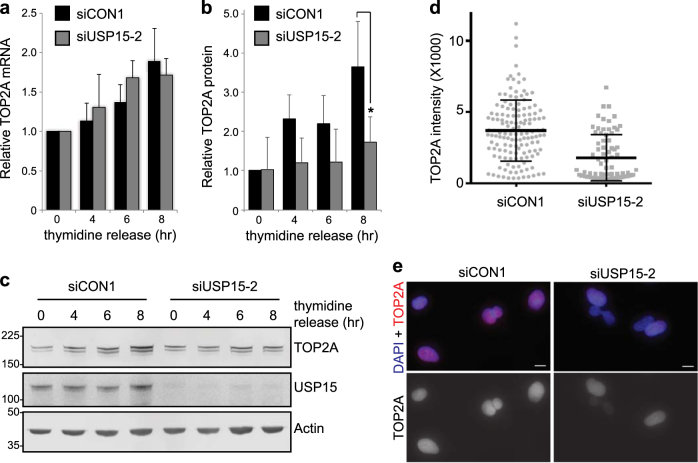
USP15 supports TOP2A accumulation in G2 as cells approach mitosis. A549 cells were transfected with either control or USP15 siRNA, arrested at G1/S by double-thymidine block and then released for the indicated times (0 h = G1/S, 4 h = S to G2, 6 h = G2, 8 h = G2 to M); RNA and protein lysates were made in parallel. **a** qRT-PCR analysis of TOP2A mRNA expression relative to actin (mean of three independent experiments, error bars SD). **b**, **c** Immunoblotting for TOP2A, USP15 and actin: **b** quantification of TOP2A protein levels (mean three independent experiments, error bars SD, paired *T*-test *P ≤ 0.05), and **c** a representative gel. **d**, **e** Immunofluorescence for TOP2A expression levels 8 h after release from double-thymidine block. Quantification of the intensity of staining for >75 cells per condition **d** (mean and SD indicated), and representative images from control and USP15 depleted cells; scale bars 10 μm **e**

As TOP2A transcription increases during G2 [41], anticipating the requirement for TOP2A in decatenation checkpoint signalling at late G2 [42, 43], we wondered whether USP15 may play a role in this temporal accumulation. In A549 cells released from G1/S thymidine arrest, TOP2A transcript and protein levels both increased during G2 as the cells approached mitosis (Fig. 2). USP15 depletion does not affect cell cycle phase distribution within an asynchronous population (Supplementary Figure S2) and did not prevent TOP2A transcription during G2 (Fig. 2a), it did, however, impede accumulation of TOP2A protein (Figs. 2b, c). Quantification of TOP2A within individual cells 8 h after release from G1/S arrest confirmed that, despite heterogeneity within the population as synchronisation diminished, cells with USP15 knockdown failed to accumulate TOP2A to the same degree as control cells (Figs. 2d, e). This role in supporting temporal accumulation of TOP2A protein during G2 is analogous to the role of USP15 in accumulation of newly synthesised REST during early G1 [34].

### USP15 is required for correct mitotic chromosome segregation

**Fig. 3:**
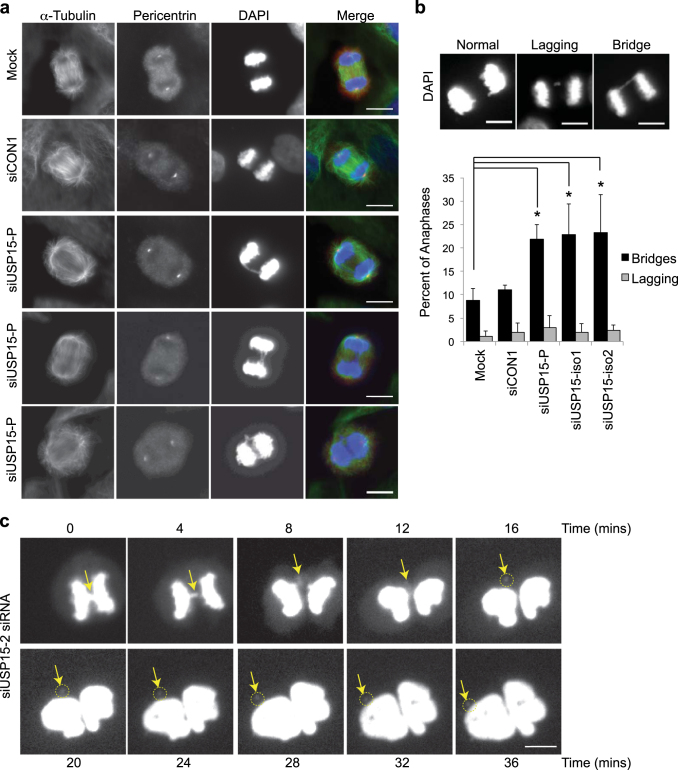
USP15 depletion causes anaphase bridges that can lead to micronuclei formation. **a**, **b** USP15 depletion in A549 cells significantly increases the number of anaphase chromosome bridges but not the number of lagging chromosomes. **a** Representative examples of anaphases observed in A549 cells, showing chromosome bridges in USP15 depleted cells. **b** Examples of each phenotype (top) with quantification of their prevalence below, data show mean of three independent experiments with >50 anaphases counted per condition per experiment (error bars SD, one-way ANOVA with Dunnett’s multiple comparison test *P ≤ 0.05). **c** Live cell imaging demonstrates that anaphase chromosome bridges (arrow) can resolve into micronuclei (circled) in USP15 depleted cells; the timecourse begins at metaphase and extends beyond cytokinesis. All scale bars are 10 μm

TOP2A catalyses transient breaking and rejoining of two strands of duplex DNA, allowing the strands to pass through one another. It accumulates at centromeres during prophase, where it is required for chromatin condensation and to decatenate intertwined sister chromatids to ensure their correct separation during anaphase [44, 45]. TOP2A depletion leads to the formation of bulky 4′,6-diamidino-2-phenylindole (DAPI)-positive anaphase bridges and ultrafine bridges, due to the persistence of catenations between sister chromatids [46–49]. We found that USP15 depletion similarly caused frequent defects in chromosome segregation during anaphase (Fig. 3a). Defects were categorised as lagging chromosomes, which fail to attach to kinetochores, or as anaphase bridges, where sister chromatids are attached to opposite spindle poles but fail to separate due to catenation (Fig. 3b). The frequency of lagging chromosomes was low in A549 cells and did not increase on USP15 depletion. However, anaphase bridges were evident in ~10% of cells exiting mitosis, and their frequency was significantly increased in cells depleted of total USP15 or of either USP15 isoform (Fig. 3b). To explore the consequence of these anaphase bridges, we followed USP15 depleted cells by time-lapse microscopy. Figure 3c shows an example of an anaphase bridge, which resolves to form a distinct micronucleus that persists after the nuclei of the daughter cells reform. Taken together with the requirement of USP15 for TOP2A accumulation as cells approach mitosis (Fig. 2), this suggests that USP15-dependent mis-segregation of chromosomes during mitosis most likely arises through defective decatenation of sister chromatids by TOP2A.

### USP15 depletion leads to centromere protein A (CENP-A)-positive micronuclei

**Fig. 4:**
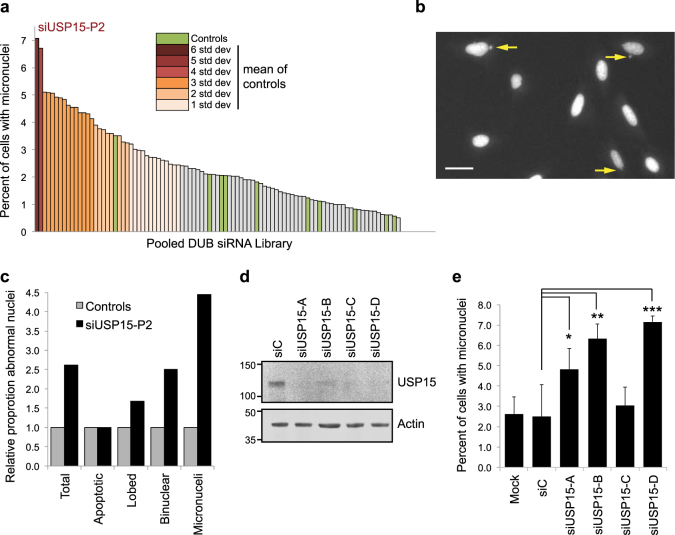
In a DUB family screen, USP15 depletion induces the most micronuclei. **a**–**c** An unbiased DUB siRNA library screen in FKHRL1-U2OS cells. The screen was performed in triplicate and >100 cells were scored for nuclear defects for each control (siC or mock-transfected) or DUB siRNA condition. **a** USP15 depletion causes most micronuclei formation. Data for the DUB siRNA library are ranked according to the percentage of cells exhibiting micronuclei, SD from the mean of the controls (green) is shown as a colour gradient. **b** Representative examples of micronuclei in USP15 depleted cells; scale bar 10 μm. **c** Formation of micronuclei is the most common nuclear defect in USP15 depleted cells; scores for the indicated categories were collated for siUSP15-P2 transfected cells in the screen. Data are shown as fold-change relative to the mean score for control conditions. **d**, **e** Multiple USP15 siRNAs cause micronuclei formation in U2OS cells. **d** A representative immunoblot showing USP15 knockdown with the individual siRNAs that comprise the library pool. **e** Quantification for the percent of cells with micronuclei, mean data from three independent experiments with >270 cells scored per condition across experiments (error bars SD, one-way ANOVA with Dunnett’s multiple comparison test *P ≤ 0.05, **P ≤ 0.01, ***P ≤ 0.005)

Independently of these experiments, we performed an unbiased screen in U2OS cells for the phenotypic effects of depleting each of the DUBs using an siRNA library, which we scored for nuclear abnormalities. Compared with the controls, depletion of USP15 led to the highest increase in formation of micronuclei among the 92 DUBs tested (Figs. 4a–c). The siRNA library comprises pools of four oligonucleotides targeting each DUB, which for USP15 were distinct from the siRNAs employed in our earlier experiments. Each of the USP15 siRNAs from the library pool effectively depleted USP15 (Fig. 4d) and three siRNAs individually recapitulated the micronuclei phenotype (Fig. 4e).

**Fig. 5:**
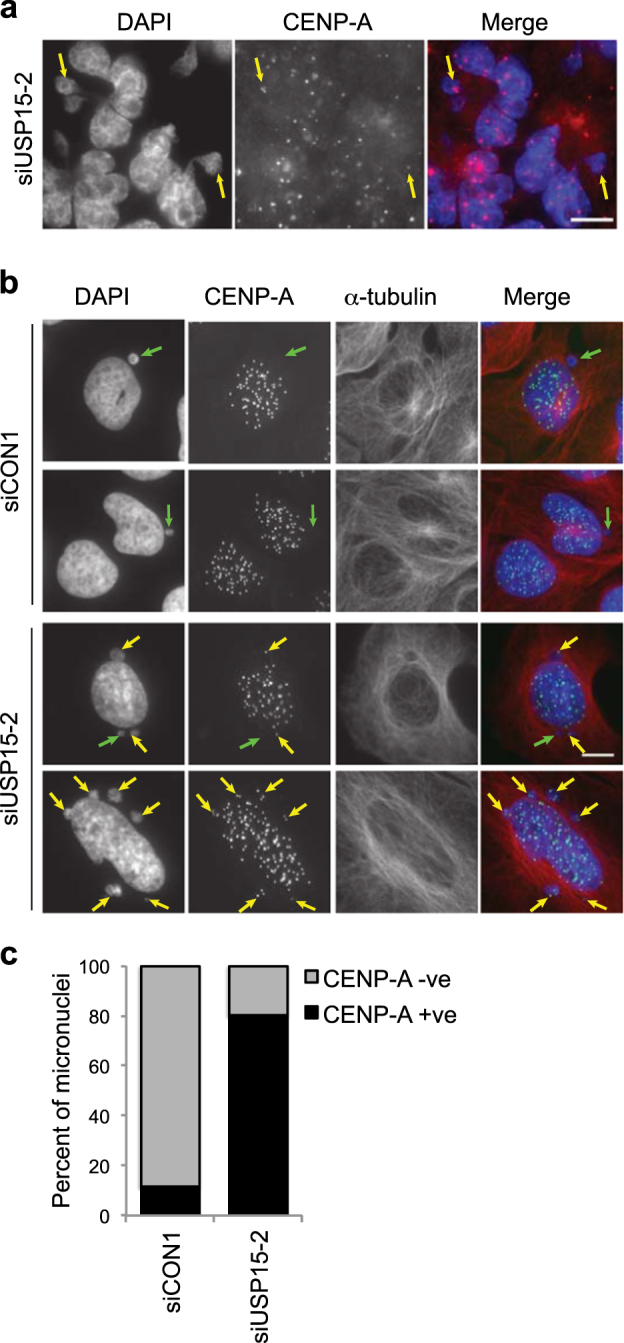
Additional micronuclei in USP15 depleted cells are CENP-A positive. Representative images of micronuclei in USP15 depleted **a** A549 cells or **b** U2OS cells, arrows indicate micronuclei that are CENP-A negative (green) or CENP-A positive (yellow); scale bar 10 μm. The percentage of micronuclei that are CENP-A positive were scored for U2OS for >80 micronuclei in each condition **c**

Micronuclei may arise because defective DNA replication or repair results in chromosome fragments or because mitotic defects such as sister chromatid catenation lead to mis-segregation of chromosomes during mitosis [49, 50]. Micronuclei arising from DNA replication or repair errors are likely to be acentric [50], whereas catenated chromosome bridges that break to from micronuclei often contain centromeres [51]. Therefore, to further characterise the observed micronuclei, we stained cells for the centromere protein CENP-A. Micronuclei positive for CENP-A were evident in USP15 depleted A549 cells (Fig. 5a) and U2OS cells (Fig. 5b). In U2OS cells transfected with non-targeting siRNA, only ~20% of micronuclei were CENP-A positive. However, in cells depleted of total USP15 with an independent siRNA, the number of micronuclei increased (Fig. 5b) and ~80% were CENP-A positive (Fig. 5c). These data further support the idea that USP15 is required to ensure correct mitotic chromosome segregation.

### Mitotic regulation of USP15 isoforms

**Fig. 6:**
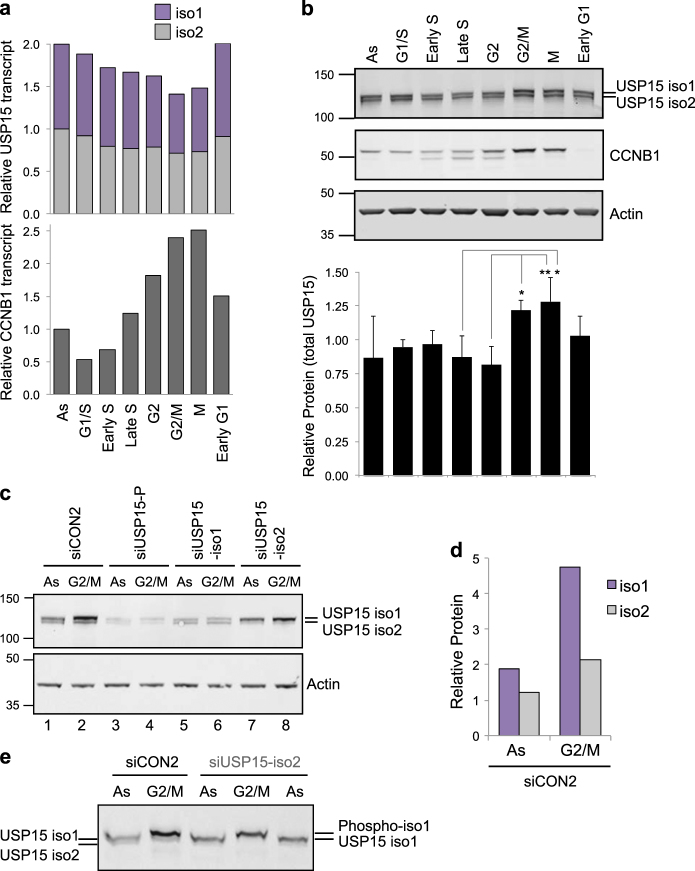
USP15 isoforms are dynamically expressed and differentially phosphorylated during the cell cycle. **a**, **b** A549 cells were synchronised using standard thymidine/nocodazole protocols to enrich for the indicated phases. **a** USP15 transcript levels remain stable during the cell cycle. Expression of USP15 splice variants and cyclin B1 (CCNB1) were quantified by qRT-PCR, mean data from three independent experiments are expressed relative to actin and normalised to the expression in asynchronous cells (As) for each splice variant. **b** USP15 protein expression levels oscillate through the cell cycle. USP15 was evaluated by immunoblotting; a representative gel is shown with quantification of total USP15 expression relative to actin below (mean of three independent experiments, error bars SD, one-way ANOVA with Tukey’s multiple comparison test *P ≤ 0.05, **P ≤ 0.01). **c**–**e** USP15 isoform-1 increases in abundance by G2/M and becomes phosphorylated. A549 cells were depleted of USP15 isoforms as indicated and protein extracts were compared by immunoblotting for asynchronous cells (As) or cells arrested at G2/M. Separation by 4–12% gradient SDS-PAGE **c** showing quantification of the siCON2 samples **d**, and analysis by Phos-tag gel electrophoresis **e**

As we have previously shown that, like TOP2A, total USP15 expression increased during G2 [34], we next asked whether both USP15 isoforms behave in the same manner. A549 cells were synchronised to enrich for populations in specific cell cycle phases to investigate how the USP15 isoforms oscillate during the cell cycle. Although their transcript levels and splice variant ratios remain relatively stable (Fig. 6a), USP15 protein levels were significantly increased by the time cells reached mitosis compared with S-phase, most noticeably for isoform-1 (Figs. 6b–e). However, depletion of either isoform had no effect on distribution between cell cycle phases in a population of cells (Supplementary Figure S2).

We previously showed that a proportion of USP15 is phosphorylated at mitosis [34], so we selectively depleted each isoform to establish whether they are differentially phosphorylated. In G2/M arrested cells, a gel mobility shift indicative of phosphorylation was seen for isoform-1 but not isoform-2 (Fig. 6b). This was confirmed by depletion of the individual USP15 isoforms (Fig. 6c) and analysis by Phos-tag electrophoresis (Fig. 6e). To determine which residues are subject to mitotic phosphorylation, we immunoprecipitated the green fluorescent protein (GFP)-tagged USP15 isoforms from A549 cells. Mass spectrometry identified two peptides that span the region encoded by exon 7 in isoform-1. One of these was phosphorylated only in mitotic extracts, mapping with high confidence to S229, the first serine in the peptide (Supplementary Figure S3). Data for the HeLa mitotic phosphoproteome also report S229 phosphorylation [37, 52]. Thus, USP15 isoform-1 is regulated independently of isoform-2, accumulating in G2 as cells approach mitosis, and subsequently undergoing S229 mitotic phosphorylation.

**Fig. 7:**
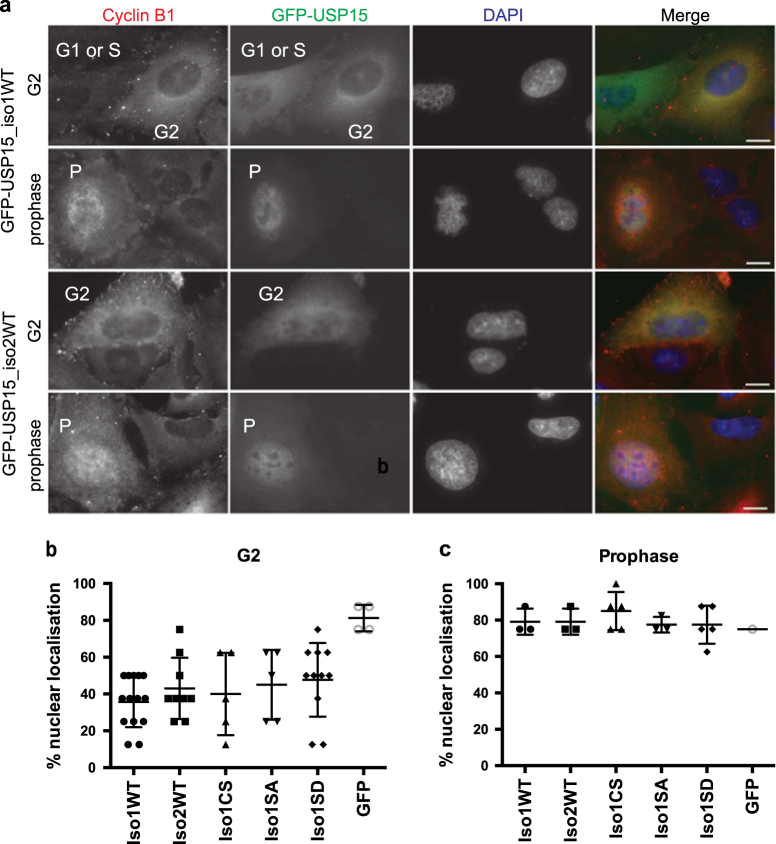
Both USP15 isoforms are predominantly cytosolic but localise to the nucleus at prophase. A549 cells were transfected with the indicated GFP-USP15 constructs or GFP only as a control. Cells were fixed and co-stained for the cell cycle phase marker cyclin B1 and DAPI, to classify as G1 or S-phase (low cytoplasmic cyclin B1), G2 (high cytoplasmic cyclin B1), or prophase (nuclear cyclin B1, uncondensed chromatin). **a** Representative immunofluorescence images of cells expressing the two wild-type (WT) USP15 isoforms in G2 cells and prophase cells; scale bars 10 μm. **b**, **c** Transfected cells were scored for relative intensity of GFP-USP15 variant expression in the nucleus compared with the cytoplasm, for cells in G2 **b** or prophase **c**; counts for >100 cells per GFP-USP15 construct were pooled from three independent experiments, mean and SD are indicated. Additional images are shown in Supplementary Figure S4

USP15 localises predominantly to the cytosol, in contrast to its closest paralogues USP4 and USP11, which, respectively, show uniform distribution and tight nuclear localisation [31]. We used GFP-tagged expression constructs to establish whether the two USP15 isoforms localise differentially, or relocalise during the cell cycle. Cells were co-stained with cyclin B1, which accumulates in the cytoplasm during G2, before translocating into the nucleus at prophase as mitosis initiates [53]. We found that GFP-USP15 localisation mirrored that of cyclin B1, being predominantly cytoplasmic in G2 cells, but localising to the nucleus at prophase (Fig. 7a). This was observed for both USP15 isoforms, was independent of catalytic activity, and was unaffected by phospho-null or phospho-mimetic mutation of S229 (Figs. 7b, c, Supplementary Figure S4). Similarly, the USP15 isoforms and phospho-mutants showed no gross difference in reactivity towards the active site-directed probe ubiquitin-vinyl methyl ester (Ub-VME) (Supplementary Figure S5). Thus, the two USP15 isoforms are similarly localised and catalytically active within cells, but both exhibit redistribution at the onset of mitosis that is independent of S229 phosphorylation. The movement of USP15 into the nucleus at prophase occurs at a time when TOP2A accumulates at centromeres to aid chromatin condensation and decatenation of sister chromatids [44, 45].

### Protection of genomic integrity and TOP2A accumulation are abrogated by S229 phosphorylation of USP15 isoform-1

**Fig. 8:**
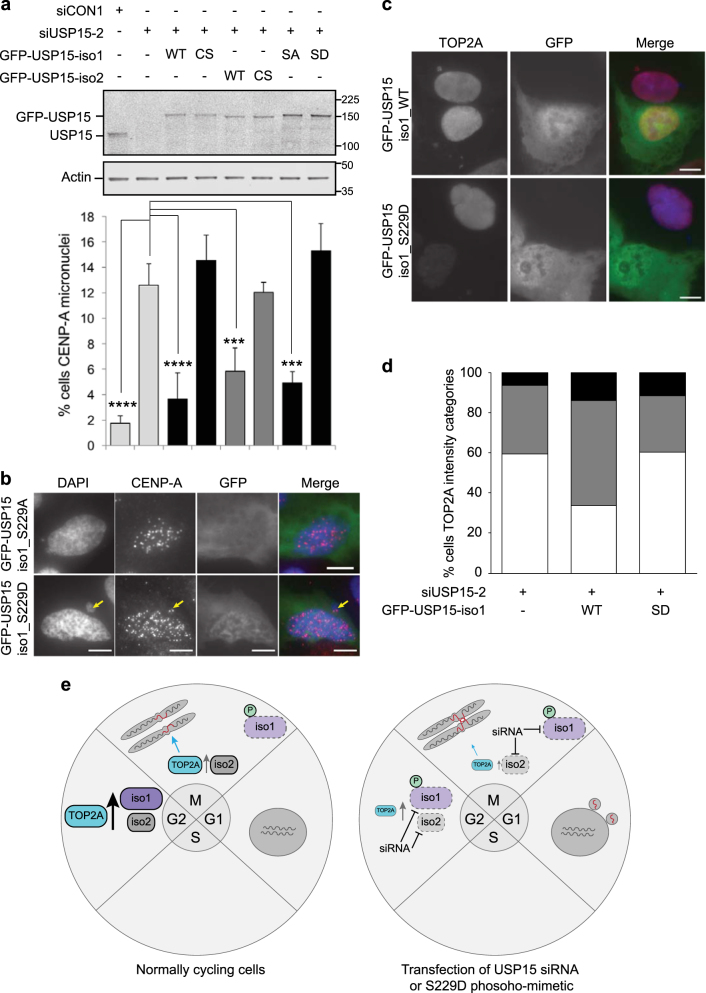
Phospho-mimetic S229D USP15 isoform-1 cannot rescue TOP2A expression or the CENP-A micronuclei phenotype. **a**, **b** Catalytic activity and non-phosphorylated S229 are required for USP15 to rescue the CENP-A micronuclei phenotype. U2OS cells were transfected with siUSP15-2 (targeting total USP15) or a non-targeting control siRNA for 72 h, the indicated siUSP15-2 resistant GFP-USP15 expression constructs were transfected for the final 24 h. **a** A representative immunoblot (top) and scoring of CENP-A-positive micronuclei from three independent experiments (below); >50 cells, with low to moderate GFP expression, were scored per condition per experiment (error bars SD, one-way ANOVA with Dunnett’s multiple comparison test ***P ≤ 0.005, ****P ≤ 0.001). **b** Representative images for cells in the phospho-null and phospho-mimetic rescue conditions. All scale bars are 10 μm. **c**, **d** Non-phosphorylated S229 is required for USP15 to rescue TOP2A expression. Experiments were performed as above in U2OS cells, and immunofluorescence intensity for TOP2A assessed following rescue with WT or S229D USP15 isoform-1, **c** representative images (scale bars are 10 μm), and **d** quantification for >100 cells per condition; relative TOP2A intensities shown as white (<1), grey (1-2) or black (>2). **e** schematic illustrating our working model for USP15 regulation of TOP2A, anaphase bridges and micronuclei

Given the premise that pervasive mitotic phosphorylation often inactivates protein function [37], we explored whether mitotic S229 phosphorylation of USP15 isoform-1 might impede its roles in supporting TOP2A accumulation, and in preventing micronuclei forming from unresolved anaphase bridges. To this end, we performed rescue experiments in U2OS cells depleted of total USP15. The appearance of CENP-A-positive micronuclei in USP15 depleted cells was reversed by exogenous expression of either USP15 isoform (Fig. 8a). This is consistent with our data showing that depletion of either USP15 isoform reduces TOP2A accumulation during G2 (Fig. 2). The catalytically inactive USP15 (C298S/C269S) isoforms could not rescue micronuclei, confirming that this role of USP15 in genome maintenance requires its DUB activity. Importantly, in contrast to the phospho-null mutant (S229A), expression of phospho-mimetic (S229D) USP15 isoform-1 did not reduce the number of CENP-A-positive micronuclei (Figs. 8a, b). Thus, S229 phosphorylation impedes the ability of USP15 isoform-1 to protect genome integrity. In contrast to the S229 phospho-mimetic, wild-type USP15 isoform-1 can rescue the phenotype, presumably as it remains unphosphorylated in G2, and can support TOP2A accumulation during this phase. Consistent with our hypothesis that USP15 affects genome stability through TOP2A, wild-type USP15 isoform-1, but not S229D phospho-mimetic USP15, increased expression levels of TOP2A in USP15 depleted U2OS cells (Figs. 8c, d).

Taken together, our data suggest that G2 accumulation of TOP2A, and its function in mitotic decatenation, are sensitive to the combined amount of the two USP15 isoforms in cells. When available USP15 is reduced, by siRNA depletion of either isoform, or by S229 phosphorylation of isoform-1, this promotes genome instability (Fig. 8e).

### USP15 isoform-1 is selectively upregulated in NSCLC

**Fig. 9:**
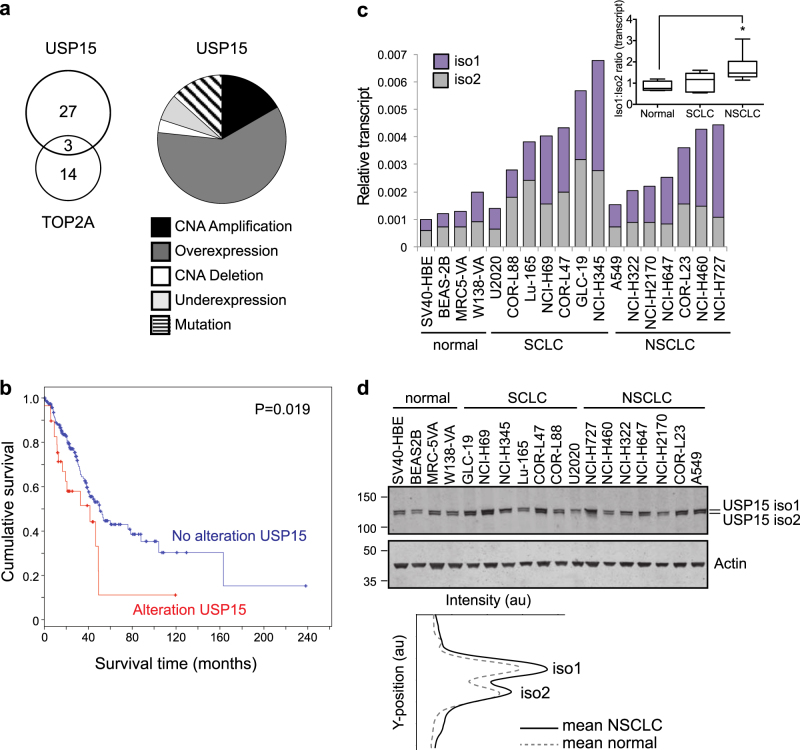
USP15 is amplified and USP15 isoform-1 is overexpressed in NSCLC. **a**, **b** Data from the TCGA provisional cohort of 230 lung adenocarcinoma patients. **a** Alterations in USP15 (*n* = 30), highlighting the frequency of mutations, copy number alterations (CNA) or transcript expression changes (right) and overlap with cases exhibiting TOP2A alterations (left). **b** Kaplan–Meier overall survival curves stratified according to alterations in USP15; log rank test *P* = 0.019 for patients with (*n* = 30) compared with those without (*n* = 200) alterations. **c**–**d** USP15 isoform-1 is selectively upregulated in NSCLC. Endogenous expression of USP15 isoform-1 and isoform-2 in a panel of 18 cell lines representing normal lung and lung cancer sub-types. **c**, qRT-PCR for splice variants; relative expression is shown normalised to actin. Inset boxplot shows the ratio of USP15 isoform-1 to 2 splice variants for each cell type: normal (*n* = 4), SCLC (*n* = 7) and NSCLC (*n* = 7); one-way ANOVA with Dunnett’s multiple comparison test *P ≤ 0.05. **d** Immunoblotting of whole-cell protein extracts for USP15 isoforms, with mean quantification of the two isoforms in normal or NSCLC cells below

Finally, we asked whether USP15 may be dysregulated in lung cancer in a way that might promote genome instability. Copy number alterations for USP15 are reported in glioblastoma, breast, ovarian, and pancreatic cancers [20, 21]. In relation to our data for A549 cells, we examined the provisional TCGA data set for lung adenocarcinoma and found that USP15 is altered in 30 of 230 cases (13%), most commonly being amplified and/or overexpressed (Fig. 9a). Interestingly, although TOP2A is modified in 17 cases (7%), there is little overlap with cases harbouring USP15 alterations (Fig. 9a). Of note, alterations in USP15 are associated with worse prognosis within this cohort of lung adenocarcinoma patients (Fig. 9b).

To explore the relative cellular abundance of the two USP15 isoforms in lung cancer, we used quantitative real-time RT-PCR (qRT-PCR) with primers that specifically detect the splice variants encoding USP15 isoform-1 or isoform-2 (Supplementary Figure S1). We quantified transcript expression across a panel of cell lines derived from normal lung, small cell lung cancer (SCLC), or non-small cell lung cancer (NSCLC), the latter being predominantly adenocarcinoma (Fig. 9c). Both splice variants were endogenously expressed in all the cell lines examined, and they accounted for equivalent proportions of the total USP15 in normal lung and SCLC cell lines. However, the overall abundance of USP15 mRNA was increased in both SCLC and NSCLC, and in the latter was mainly attributable to the transcript variant encoding isoform-1 (Fig. 9c, inset). Broadly speaking, these transcript expression profiles concur with the relative abundance of the two USP15 isoforms on immunoblotting, with isoform-1 more abundant in NSCLC than the normal lung cell lines (Fig. 9d). Together these data suggest that increased expression of USP15 in lung adenocarcinoma is biased towards higher expression of isoform-1, which once S229-phosphorylated is less able to protect against micronuclei formation through mitotic mis-segregation.

## Discussion

Here we report TOP2A as a novel USP15 regulated protein, revealing a new role for USP15 in guarding genome integrity. In addition, we describe the first example of isoform-specific DUB phospho-regulation, as we show that mitotic phosphorylation of S229 selectively abrogates this role for USP15 isoform-1. USP15 and its most closely related DUBs, USP4, and USP11, have been implicated in other aspects of genome integrity at the level of DNA repair [54–56], but here we identify a distinct role for USP15 in G2/M decatenation by TOP2A.

TOP2A expression increases during G2 and decreases when cells complete mitosis. It is essential for the decatenation checkpoint in late G2, and for resolving intertwined sister chromatids and facilitating chromatin condensation at the onset of mitosis, thus ensuring accurate division of the genome during anaphase [42–45]. We show that G2 accumulation of TOP2A and its function in mitotic decatenation are sensitive to the total amount of cellular USP15, which if limited by siRNA depletion or S229 phosphorylation, results in genome instability (Fig. 8e).

Our data also shed new light on temporal regulation for the two major USP15 isoforms during the cell cycle. The alternative splicing mechanism that generates these isoforms remains unclear, although VEZF1-dependent RNA pol II pausing is reported to promote exon inclusion favouring isoform-1, whereas the polypyrimidine tract binding protein PTB promotes exon skipping to favour isoform-2 [57]. USP4, the closest homologue of USP15, is also alternatively spliced in a similar fashion, although the skipped amino-acid cassette differs in sequence to that in USP15, and the two USP4 isoforms are proposed to act in different cellular compartments [58].

The USP15 isoforms were historically studied interchangeably, although an example of isoform-dependent substrate specificity was recently reported [18]. In contrast, the USP15 role that we describe here, can be performed by either isoform. Importantly though, we show for the first time that the USP15 isoforms are differentially regulated during the cell cycle, with S229 phosphorylation specifically abrogating the role of USP15 isoform-1 in regulating TOP2A expression and preventing micronuclei formation. In cycling cells, endogenous USP15 isoform-1 remains unphosphorylated during G2 when it is required for TOP2A accumulation. S229 phosphorylation occurs as cells enter mitosis, at around the time USP15 relocalises to the nucleus and TOP2A localises to centromeres to perform its role in decatenation. In our experiments (Fig. 8), exogenously expressed USP15 isoform-1 can support G2 accumulation of TOP2A and this appears sufficient to rescue the micronuclei phenotype. In contrast, activity of the S229D phospho-mimetic towards TOP2A is lacking not only during mitosis, but also in G2, rendering it unable to rescue either the TOP2A or micronuclei phenotypes.

The only kinases described to date for USP15 are ATM, which phosphorylates the two isoforms at S678/S649, respectively [39], and CK2 that targets undetermined residues [36]. There is no strong kinase consensus around S229 and the various prediction algorithms suggest a number of candidates, including CK1, GS3K, and ERK. The kinase that phosphorylates USP15 at S229 remains to be experimentally determined.

Post-translational modifications including ubiquitylation are important regulators of TOP2A function [59] and E3 ligases for TOP2A include BRCA1 [60], SCF^FBXW7^ [61], and RNF168 [62]. Similarly, in addition to our data for USP15, USP10 [62], and OTUD3 [63] were recently reported as DUBs that regulate TOP2A. Such apparent redundancy may be essential to regulate critical checkpoint proteins. A notable example is P53, which is regulated by multiple DUBs acting at different cell cycle stages, or in different cellular compartments, to co-ordinate fine-tuned, temporal control over P53 expression (reviewed in Darling et al. [10]). OTUD3, a predominantly cytosolic DUB [31], is proposed to regulate TOP2A specifically when complexed with the tumour suppressor PTEN [63]. In contrast, USP10, also a cytosolic DUB [31], deubiquitylates TOP2A in complex with the E3 ligase RNF168 [62], which is also important in DNA damage response signalling for double-strand break repair [64]. Thus, a suite of E3 ligases and DUBs may regulate TOP2A in specific contexts to fine-tune TOP2A function in response to cellular needs.

Importantly, we also demonstrate that both isoforms of USP15 are commonly endogenously expressed in cells, whereas isoform-1 expression is preferentially upregulated in NSCLC cell lines. Interestingly, USP15 amplification is reported in certain cancers [20], and here we show this is also the case for lung cancer, where elevated USP15 is associated with worse prognosis in clinical lung adenocarcinoma (Fig. 9). However, in NSCLC cell lines, we find the USP15 isoform-1 is preferentially upregulated. As USP15 has multiple verified and candidate substrates, it is difficult to assess the relative importance for regulation of TOP2A in USP15 amplified and/or USP15 isoform-1 overexpressing cancer cells. However, we postulate that such an imbalance, in favour of isoform-1 that can be mitotically inactivated by phosphorylation, may dampen the temporal role of USP15 in promoting genome stability through TOP2A, while not impacting on the other roles for USP15 in regulating alternative oncogenic pathways.

## Materials and methods

### Cell culture

A549 and U2OS cells (ECACC) were cultured in high glucose Dulbecco’s modified Eagle’s medium (DMEM) supplemented with 10% foetal bovine serum (FBS) and 1% non-essential amino acids. All other lung cancer cell lines (sourced as previously described [65]) were cultured in RPMI supplemented with 10% FBS. FKHRL1-U2OS cells (Thermo Scientific, Bioimage Products, Lafayette, USA) were maintained in high glucose DMEM supplemented with 10% FBS, 100 units/ml penicillin/streptomycin and 0.5 mg/ml geneticin. All cells were cultured in a humidified incubator at 37 °C and 5% CO_2_. Cell lines were authenticated by short tandem repeat (STR) profiling, verified as mycoplasma free and cultured for limited passage numbers.

### Cell synchronisation

To obtain cell populations synchronised at G1/S or in early S, late S, or G2, A549 were subject to double-thymidine block (2 mM thymidine (Sigma) for 18 h, released into fresh media for 8 h, arrested with thymidine for 17 h) then lysed directly or released into full medium for 2.5, 5.5, or 7.5 h. To obtain cell populations synchronised at G2/M or in M, or early G1, A549 were subject to a thymidine/nocodazole block (2 mM thymidine for 24 h, released into full medium containing 100ng/ml nocodazole (Sigma) for 14 h) then mitotic cells were collected by knocking off the dish, and either lysed directly or replated into full medium for 0.5 or 4 h. Propidium iodide staining with flow cytometry analysis, and immunoblotting for a panel of stage-specific cell cycle markers, are routinely used to confirm enrichment of cell cycle phases. For analysis of each timepoint, adherent cells were trypsinised and pooled with non-adherent cells from the medium, and then processed for RNA or protein extraction.

### RNA interference

A549 or U2OS cells were seeded at 6 × 10^4^ cells per well in six-well plates and transfected the following day with 40 nM siRNA using Oligofectamine (Invitrogen); cells were analysed 72 h later by immunoblotting or imaging. For rescue experiments, 48 h after siRNA transfection, U2OS cells were transfected with 1 μg of plasmid using GeneJuice (Novagen) and processed for immunofluorescence 24 h later. siGenome siRNAs (Dharmacon) that target both of the USP15 isoforms were used as a pool named siUSP15-P (siUSP15-1 (D-006066-01), siUSP15-2 (D-006066-02), and siUSP15-17 (D-006066-17)). siGenome non-targeting control siRNAs were siCON1 (D-001210-01) and siCON2 (D-001210-02). siGenome custom siRNAs specific for each USP15 isoform were: siUSP15-iso1 (5’-ACAACAUGAACAACAGAAAUU-3’) and siUSP15-iso2 (5’-CUUUCUACUCCUAAUGUGAUU-3′).

A custom-designed DUB siRNA library consisting of four pooled oligos for each of 92 human DUBs (Qiagen) was used for the RNAi screen. Individual Qiagen siRNAs targeting both USP15 isoforms were used for follow-up studies (siUSP15-A (SI00087353), siUSP15-B (SI00087360), siUSP15-C (SI00087367), siUSP15-D (SI03072909)), either alone or as a pool (USP15-P2), together with the All-Stars negative control siRNA (1027281, siC). For the siRNA library screen, FKHRL1-U20S cells were seeded at 3000 cells per well in 96-well plates and reverse transfected with 20 nM siRNA using Lipofectamine RNAiMax (Life Technologies). Forty-eight hours after transfection, medium was replaced with HBSS (Gibco) containing 2.5 µM DRAQ5 (Biostatus) and the cells then imaged.

### Plasmid DNA constructs

USP15 isoform-1 (NM_001252078.1) and USP15 isoform-2 (NM_006313.2) were cloned using the Gateway system into pDONR233 entry constructs. siRNA-resistant (to siUSP15-2), catalytically inactive and phospho-mutant forms of USP15 were generated by Quikchange site-directed mutagenesis in pDONR233 using complementary primer pairs: USP15-siRes2, 5′-CCATGAAAAAAGAACGCAC**T**TT**A**GA**G**GT**A**TACTTAGTTAGAATG-3′, USP15 isoform-1 (C298S) or USP15 isoform-2 (C269S), 5′-GTAACTTGGGAAATACG**A**GTTTCATGAACTCAGC-3′, USP15 isoform-1 phospho-null (S229A) 5′-CTTCTACTCCTAAG**G**CCCCAGGTGCATCC-3′ and phospho-mimetic (S229D) 5′-CTTCTACTCCTAAG**GA**CCCAGGTGCATCC-3′. Each construct was sequence verified and shuttled into expression vectors. H2B-Cherry was a gift from Stephen Royle, University of Warwick, UK.

### RNA extraction and real-time PCR

Total RNA was extracted using RNeasy columns (Qiagen) and complementary DNA was reverse transcribed from 1 μg RNA with RevertAid H-minus M-MuLV reverse transcriptase (Fermentas) using an oligo-dT primer (Promega). qRT-PCR was performed in triplicate using SYBR Green supermix and a CFX real-time PCR detection system (Bio-Rad). Primer sequences were: ACTB (for: 5′-CACCTTCTACAATGAGCTGCGTGTG-3′, rev: 5′-ATAGCACAGCCTGGATAGCAACGTAC-3′), total USP15 (for: 5′-CAGACAGCACCATTCAGGATGC-3′, rev: 5′-GAGTTTTTCACATTAGGAGTAG-3′), USP15 isoform-1 (for: 5′-CAGACAGCACCATTCAGGATGC-3′, rev: 5′-AAAATTGGATGCACCTGGGGAC-3′), USP15 isoform-2 (for: 5′-CAGACAGCACCATTCAGGATGC-3′, rev: 5′- GAGTTTTTCACATTAGGAGTAG-3′), cyclin B1 (for: 5′-GCTCTTCTCGGCGTGCTGC-3′, rev: 5′- CCTGCCATGTTGATCTTCG-3′), TOP2A (for: 5′-TGAAAACCCAACCTTTGACTC-3′, rev: 5′- GCTTTCTACAATACCACAGCC-3′). Samples underwent two-step amplification at 94 °C (30 s) and 60 °C (60 s); melt curves were analysed after 40 cycles. The Ct values for test genes were normalised to ACTB and relative expression represented as 2^−[ΔCt]^ for the cell panel or 2^−[ΔΔCt]^ relative to a comparator sample in experiments.

### Antibodies

Antibodies used were mouse anti-USP15 (H00009958-M01, Abnova), anti-TOP2A (sc-165986, Santa Cruz), anti-cyclin B1 (05-373, Millipore), anti-β-actin (ab6276, Abcam), anti-α-tubulin (DM1A, Sigma), and anti-CENP-A (ab13939, Abcam); rabbit anti-actin (A2066, Sigma), anti-pericentrin (ab4448, Abcam). Polyclonal affinity-purified sheep anti-GFP was generated in house (IAP).

### Immunofluorescence and microscopy

Cells were fixed in 4% paraformaldehyde, quenched with ammonium chloride, and permeabilised with 0.1% triton prior to blocking and primary antibody incubation. Alexa-Fluor 488 and Alexa-Fluor 594 coupled secondary antibodies (Molecular probes) were used. Coverslips were mounted on Moviol supplemented with DAPI at 1:10,000. Cells were imaged using a Nikon Eclipse Ti (CFI Plan Apochromat 40 × N.A. 0.95, W.D. 0.14 mm) microscope. For TOP2A quantitation, nd2 files were opened in FIJI and the DAPI channel used to create regions of interest, which were overlaid on the red channel to measure the mean TOP2A intensity per cell. Micronuclei rescue experiment slides were scored blind, with the identity of samples anonymised until after scoring was complete. For live cell imaging, an Okolab incubator maintained cells at 37 °C, 5% CO_2_ and CFI Plan-Fluor 10X N.A.0.3 or CFI Super-Plan-Fluor 20X N.A.0.45 objectives were used.

### Immunoblotting

Whole-cell extracts were prepared by direct addition of hot Laemmli buffer and incubation at 110 °C for 10 min with intermittent vortexing. Following bicinchoninic acid assay (Thermo Scientific), equivalent amounts of proteins were resolved by sodium dodecyl sulfate–polyacrylamide gel electrophoresis (SDS-PAGE) and transferred to BiotraceNT membrane (VWR) for incubation with primary antibodies. To accentuate band shifts caused by phosphorylation, samples were run on 6% polyacrylamide gels with 20 µM Phos-Tag Acrylamide (NARD institute, AAL-107) and 40 µM MnCl_2._ Proteins were visualised using donkey anti-mouse, anti-rabbit, or anti-sheep secondary antibodies conjugated to the IRDyes IR680-LT, or IR800 (LI-COR), and a LI-COR Odyssey 2.1 system, with 16-bit images quantified in ImageStudio.

### Mass spectrometry

SILAC-labelled A549 cells were transfected with siUSP15-P or siCON1 for 72 h prior to analysis. Whole-cell protein lysates were separated on NuPAGE Novex 4–12% Bis-Tris Gels (Invitrogen). Gel slices were chopped into cubes <1mm^3^ and de-stained with 50% acetonitrile/50% 100 mM ammonium bicarbonate. Samples were reduced (10 mM DTT, 56 °C, 1 h) and alkylated (50 mM iodoacetamide, 30 min, room temperature). After dehydration with acetonitrile, gel pieces were incubated with mass spectrometry grade Trypsin Gold (Promega) at 10 ng/μl (18 h, 37 °C). Peptides were extracted by incubation in acetonitrile, followed by two 1% formic acid incubations, and a final acetonitrile extraction. Samples were dried by rotary evaporation in a Speedvac and peptides re-suspended in 1% formic acid.

Peptides were separated using a nanoACQUITY UPLC system (Waters), coupled to an LTQ Orbitrap XL mass spectrometer (Thermo Scientific) with a Proxeon nano-electrospray source. In all, 5 μl of the digest was injected into a 180 μm × 20 mm, 5 μm C18 symmetry trapping column (Waters) in 0.1% formic acid at 10 μl/min before being resolved on a 25 cm × 75μm BEH-C18 column (Waters), in an acetonitrile gradient in 0.1% formic acid, with a flow rate of 400 nl/min. Full scan MS spectra (*m/z* 350–2000) were generated at 30,000 resolution, ions fragmented by collision-induced dissociation (collision energy 35%, 30 ms) and subjected to MS/MS in the linear quadrapole ion trap. All spectra were acquired using Xcalibur software (version 2.0.7; Thermo Fisher Scientific). RAW files were analysed using Maxquant version 1.4.1.2.

### Bioinformatics and statistical analysis

The provisional TCGA data set for lung adenocarcinoma was accessed and analysed using cBioportal [66, 67]. Biochemical measurements represent several thousand cells; these data are represented as the mean value from at least three independent experiments, with error bars showing standard deviation (as indicated in Figure legends). No statistical method was used to predetermine sample size. For expression levels or phenotypes of individual cells, where feasible, we aimed to count/score at least 100 cells per condition, except where the experiment precluded this. All statistical tests for experimental data were performed using GraphPad Prism version 6.00 for Mac; *P*-values <0.05 were considered to be significant. Data were analysed by *T*-test or analysis of variance as appropriate (indicated in Figure legends). These parametric tests are suitable for continuous data sets without off-scale measurements, and assume Gaussian distribution, which was confirmed by the D’Agostino & Pearson omnibus normality test in Prism, and equivalent variance between samples confirmed by a variance homogeneity test.

## Electronic supplementary material

Supplementary information
