# Supplementary material for: The deubiquitylase USP15 regulates topoisomerase II alpha to maintain genome integrity
Source: Oncogene. 2018 Feb 12;37(17):2326–42. doi: 10.1038/s41388-017-0092-0 (PMC5916918; doi:10.1038/s41388-017-0092-0)
Supplement: Supplementary file 1 — Supplementary information [file 41388_2017_92_MOESM1_ESM.pdf]

Supplementary Material

Supplementary Methods

Cell cycle experiments

Asynchronous A549 cells were transfected with siRNAs and 72hr later were fixed in ethanol prior to propidium iodide staining to determine DNA content. The fluorescence of single cells was quantified by flow cytometry using a BD FACScaliburTM system and WinMDI software. For mitotic counts, asynchronous A549 cells were seeded onto coverslips, transfected with siRNAs for 72hr, then stained for tubulin and DAP1. The percentage of cells in mitosis was scored using a Nikon Eclipse Ti (CFI Plan Apochromat 40× N.A. 0.95, W.D. 0.14mm) microscope.

Ub-VME binding assays

Cells were lysed in non-denaturing buffer (50mM Tris pH: 7.5, 5mM $MgCl_2$, 250mM sucrose,1mM DTT, 2mM ATP) on ice and homogenized by progressively passing through 23G, 26G and 30G needles with PhosStop (Roche) added immediately. Lysates were cleared by centrifugation (20min, 14,000rpm, 4°C), a BCA assay performed and protein concentrations standardized to 0.5mg/ml. 15µg protein was incubated with 75ng Ha-Ub-VME (UbiQ) for at 37°C with shaking at 300rpm. 5x sample buffer (15% SDS, 50% Glycerol, 16% 2-Mercaptoethanol, 0.5% Bromophenol Blue, 312.5mM Tris pH 6.8) was added and samples heated at 95°C for 5min to terminate the reaction before analysis by immunoblotting.

Immunoprecipitation of GFP-USP15 constructs from synchronized cells for mass spectrometry

A549 cells were transfected with GFP-USP15 plasmids and synchronized at G1/S or G2/M, as described in main materials and methods. Cells were lysed in NP-40 buffer (25mM Tris pH 7.5, 100mM NaCl, 50mM NaF, 0.5 % (w/v) NP-40, 2mM $MgCl_2$, 1mM EGTA, Complete Mammalian Proteases Inhibitors (Roche) and PhosStop (Roche)) with rocking on ice for 15min. The protein concentration of each sample was determined after suitable dilution using a Bicinchoninic Acid (BCA) assay (Thermo Scientific) and 2mg lysate used for each immunoprecipitation. An anti-GFP single chain antibody (GFP-nanotrap) was coupled to sepharose beads, added to lysates and incubated at 4°C overnight with rotation. Beads were pelleted and washed three times in Wash buffer 1 (25mM Tris pH7.5, 50mM NaCl, 50mM NaF, 0.05% (w/v) NP-40, 2mM $MgCl_2$ with mammalian protease inhibitors and PhosStop) then once

in Wash buffer 2 (10mM Tris pH 7.5, 2mM $MgCl_2$). The beads were boiled for 5min with intermittent vortexing in 2% SDS to elute bound proteins. Prior to running on a gel, samples were reduced (boiled 2min with 10mM DTT) and alkylated (chloroacetamide added to 50mM for 30min, room temperature in dark). The immunoprecipitation eluate was separated on NuPAGE Novex 4–12% Bis-Tris Gels (Invitrogen), the band corresponding to GFP-USP15 cut out and processed for mass spectrometry as described in the main materials and methods, but omitting reduction and alkylation during the in gel-digest and using (m/z 300–2000).

Supplementary Figure Legends

Supplementary Figure S1. Expression of USP15 splice variants and isoforms in A549 cells.

a, Schematic of the USP15 isoforms (top), showing the 28 amino acid serine-rich cassette unique to isoform 1, and the alternative splice variants that encode them (below) illustrating the position of isoform-specific primers and siRNAs. **b & c,** Independent depletion of the USP15 isoforms. A549 cells were transfected for 72hr with siRNAs targeting isoform-1, isoform-2, or both USP15 isoforms (USP15-P). (**b**) Total RNA was analysed by qRT-PCR for the two USP15 splice variants, data are normalised to actin and relative to the non-targeting siRNA siCON2. (**c**) Whole cell protein extracts were subject to immunoblotting for USP15, a representative experiment is shown with quantitation of the two isoforms (below).

Supplementary Figure S2. USP15 depletion does not alter cell cycle distribution.

a, Flow cytometry analysis of A549 cells 72hr post siRNA transfection. Single cells were gated based on propidium iodide intensity; histogram shows the distribution of the cell population between cell cycle phases. **b,** The mitotic index was counted for A549 cells 72hr post siRNA transfection.

Supplementary Figure S3. Mitotic S229 phosphorylation of USP15 isoform-1.

Schematic shows the experimental strategy to identify phosphorylated residues. GFP-USP15-iso1 or GFP-USP15-iso2 were transfected into A549 cells and protein extracts from cells arrested at either G1/S or G2/M were immunoprecipitated using a GFP-nanotrap. Immunoprecipitated proteins were compared by immunoblotting (right) or processed for mass spectrometry (left). All identified USP15 peptides, and the single phosphopeptide identified at G2/M, are shown mapped to isoform-1.

Supplementary Figure S4. USP15 S229 phospho-mutants localise similarly to wild-type USP15 isoform-1.

A549 cells were transfected with the indicated GFP-USP15 constructs or GFP only as a control. Cells were fixed and co-stained for the cell cycle phase marker cyclin B1 and DAPI, to classify cells as G1 or S-phase (low cytoplasmic cyclin B1), G2 (high cytoplasmic cyclin B1) or prophase (nuclear cyclin B1, uncondensed chromatin). Representative immunofluorescence images of cells expressing the USP15 isoform-1 C298S catalytic mutant, or the S229 phospho-mutants in G2 cells and prophase cells; scale bars 10μm. Transfected cells were scored for relative intensity of GFP-USP15 variant expression in the nucleus compared to the cytoplasm, illustrated in Figure 7.

Supplementary Figure S5. Both USP15 isoforms are catalytically reactive.

Detergent-free extracts from A549 cells were incubated with the ubiquitin active site directed probe Ub-VME at a ratio of 200:1 protein:probe for the indicated time. Analysis by immunoblotting shows an 8kDa gel mobility shift for reactive USP15 that has bound to Ub-VME. **a,** Endogenously expressed isoform-1 and isoform-2 are both reactive towards Ub-VME. **b**, Phospho-mimetic and non-phosphorylatable forms of USP15 isoform-1 are catalytically reactive towards Ub-VME. A549 cells were transfected with the indicated USP15 isoform constructs: wild-type (WT), catalytically inactive (C298S or C269S) or phospho-mutant forms of isoform 1 (S229A, S229D), and extracts were incubated with Ub-VME for 15min.

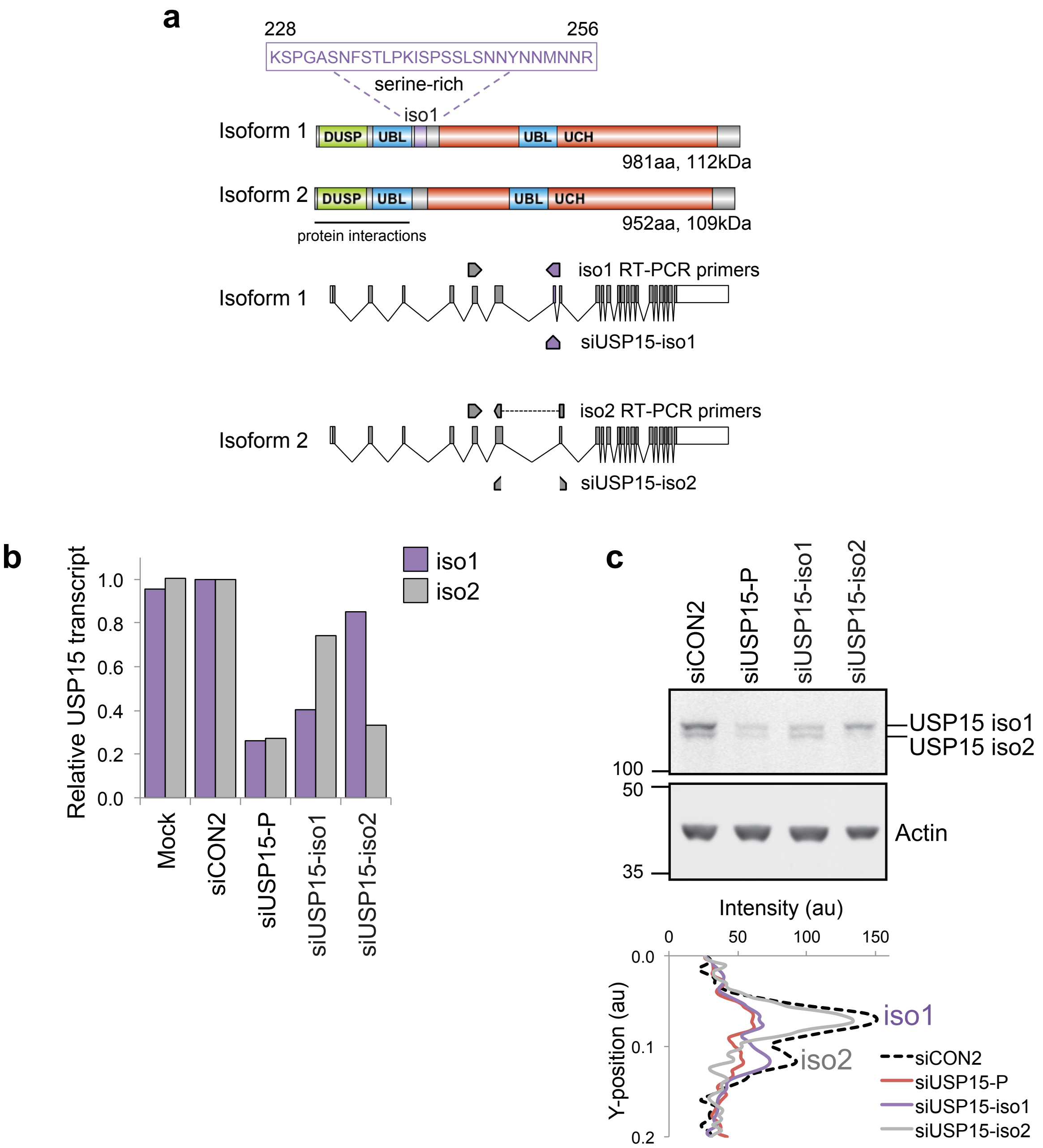

Supplementary Figure S1

a

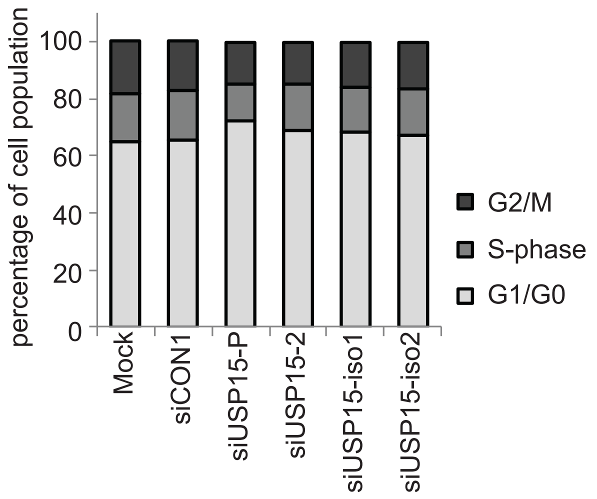

b

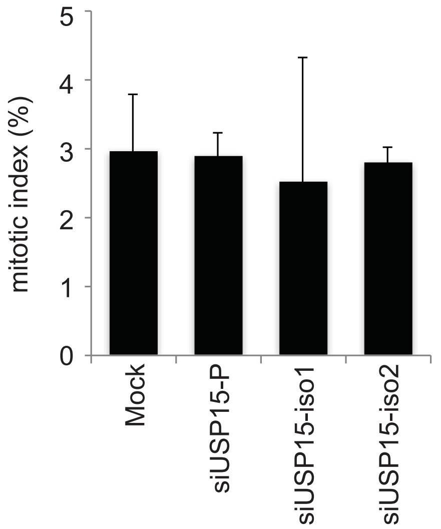

Supplementary Figure S2

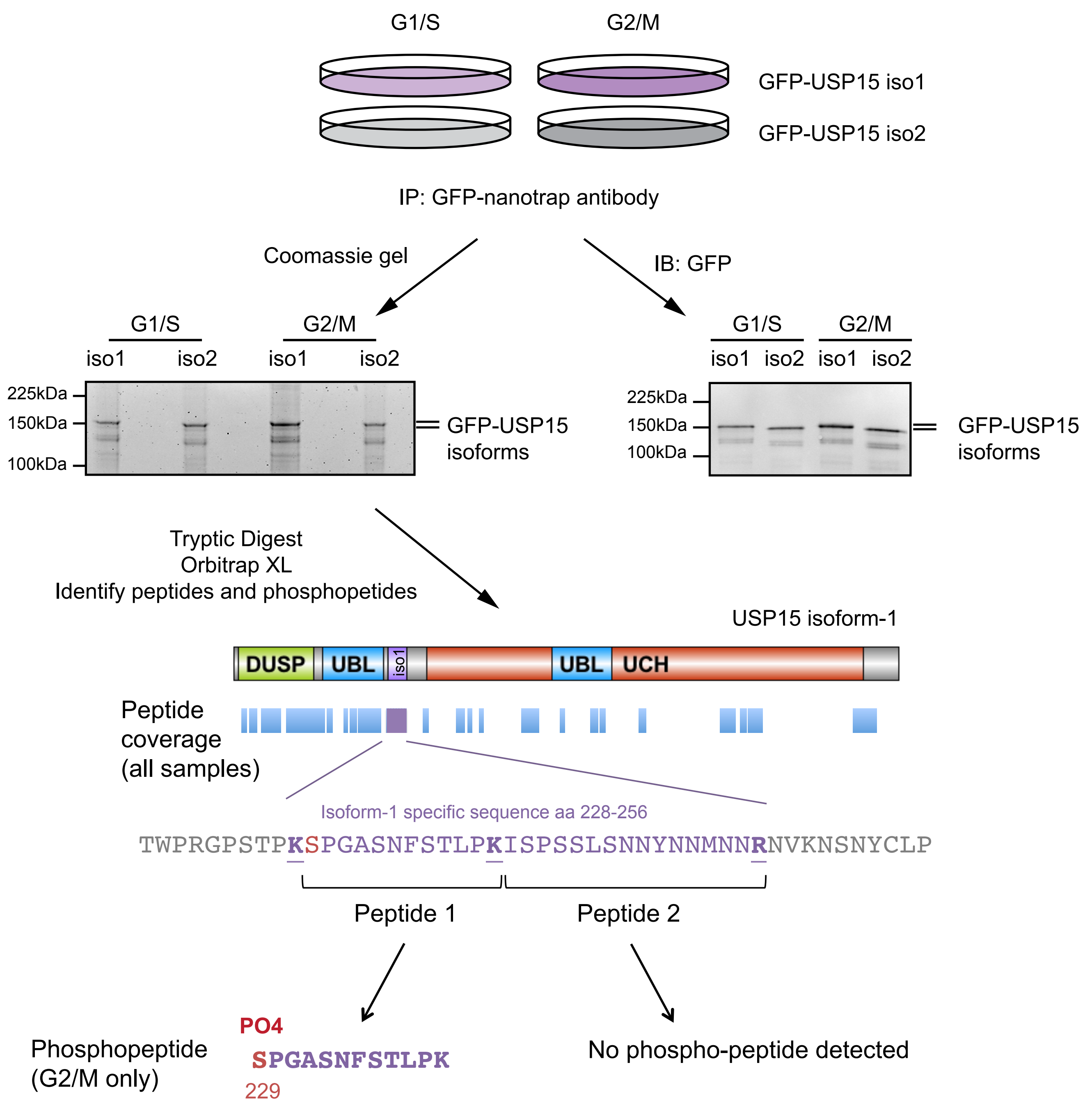

Supplementary Figure S3

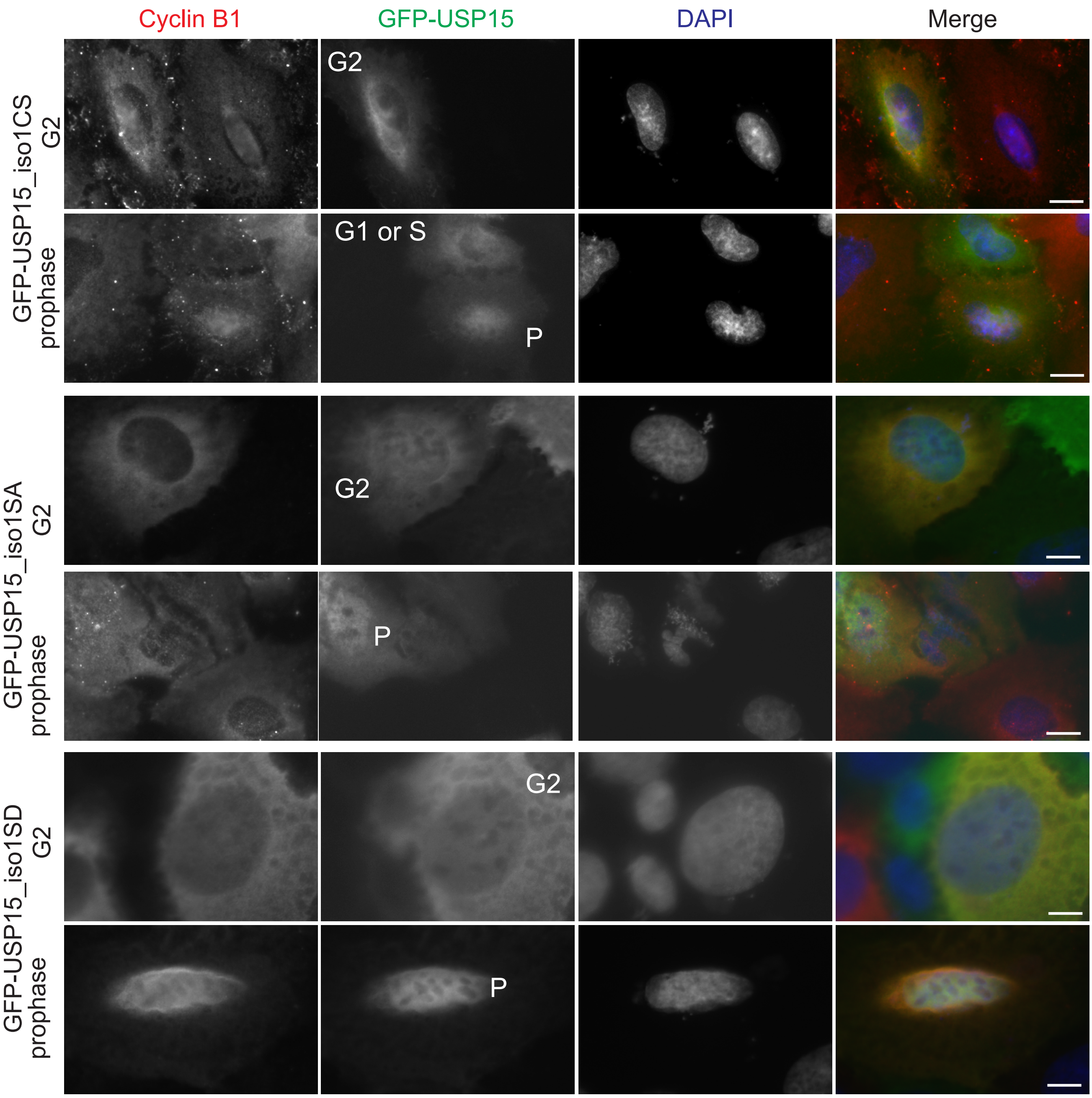

Supplementary Figure S4

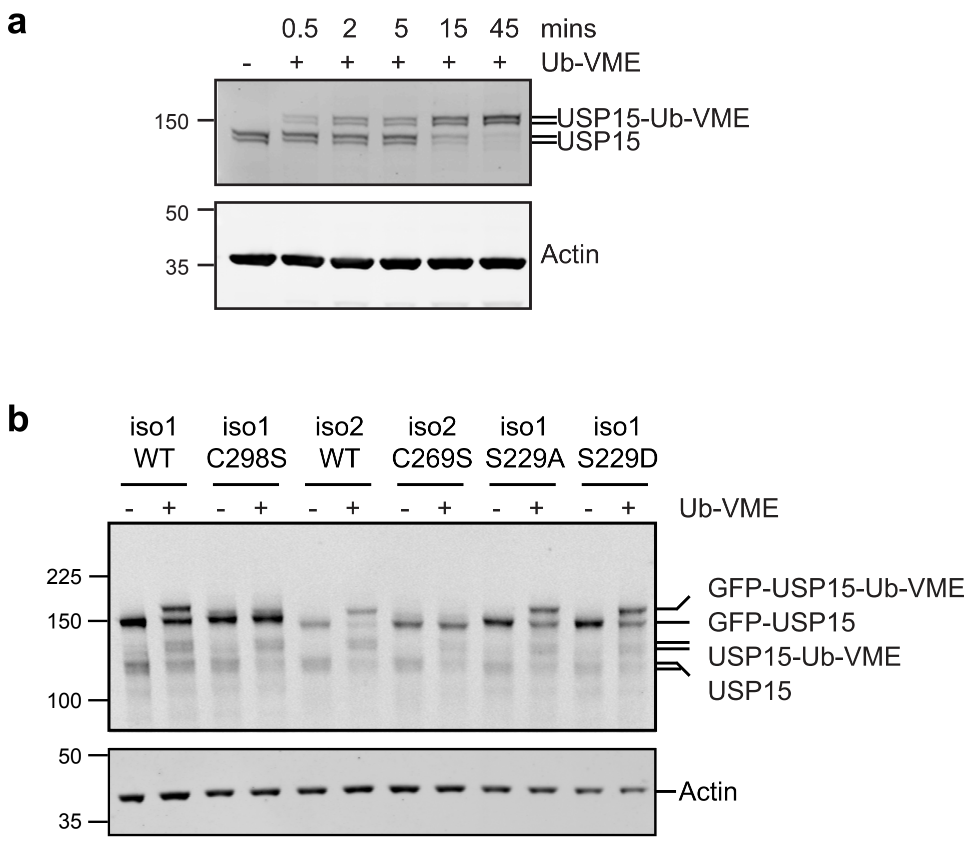

Supplementary Figure S5
